# *GATA4* screening in Iranian patients of various ethnicities affected with congenital heart disease: Co‐occurrence of a novel de novo translocation (5;7) and a likely pathogenic heterozygous *GATA4* mutation in a family with autosomal dominant congenital heart disease

**DOI:** 10.1002/jcla.22923

**Published:** 2019-05-22

**Authors:** Samira Kalayinia, Majid Maleki, Hassan Rokni‐Zadeh, Majid Changi‐Ashtiani, Hassan Ahangar, Alireza Biglari, Tina Shahani, Nejat Mahdieh

**Affiliations:** ^1^ Department of Genetics and Molecular Medicine, School of Medicine Zanjan University of Medical Sciences (ZUMS) Zanjan Iran; ^2^ Cardiogenetics Research Center, Rajaie Cardiovascular Medical and Research Center Iran University of Medical Sciences Tehran Iran; ^3^ Department of Medical Biotechnology, School of Medicine Zanjan University of Medical Sciences (ZUMS) Zanjan Iran; ^4^ School of Mathematics Institute for Research in Fundamental Sciences (IPM) Tehran Iran; ^5^ Department of Cardiology Mousavi Hospital, Zanjan University of Medical Sciences (ZUMS) Zanjan Iran; ^6^ Department of Cardiology, School of Medicine Zanjan University of Medical Sciences (ZUMS) Zanjan Iran

**Keywords:** congenital heart disease, *GATA4* gene, karyotyping, whole‐exome sequencing

## Abstract

**Background:**

Congenital heart disease (CHD) is the most common birth defect and a major health problem around the world. However, its exact etiology has remained unclear. Among various genetic contributing factors, *GATA4* transcription factor plays a significant role in the CHD pathogenesis. In this study, *GATA4* coding sequence was screened in Iranian patients of various ethnicities.

**Methods:**

Sixty six individuals with familial CHD referred to our center were recruited in this study. After receiving written informed consent from each individual or their parents, chromosomal analyses and *GATA4* variant screening were performed. Pathogenicity of the suspected variants was evaluated using available online software tools: CADD, Mutation Taster, SIFT, and PolyPhen‐2.

**Results:**

A total of twelve *GATA4* variants were detected including five intronic, 2 exonic and 3 polymorphisms as well as 2 missense mutations, the c.1220C>A and c.1309G>A. Unlike the c.1220C>A, the likely pathogenic heterozygous c.1309G>A has not been previously associated with any phenotype. Here, we not only report, for the first time, a c.1309G>A‐related CHD, but also report a novel de novo balanced translocation, 46,XY,t(5;7)(qter13;qter11), in the same patient which may have influenced the disease severity.

**Conclusion:**

From screening *GATA4* sequence in 66 Iranian patients of various ethnicities, we conclude that cytogenetic analysis and PCR‐direct sequencing of different candidate genes may not be the best approach for genetic diagnosis in CHD. Applying novel approaches such as next‐generation sequencing (NGS) may provide a better understating of genetic contributing factors in CHD patients for whom conventional methods could not reveal any genetic causative factor.

## 1 INTRODUCTION

Congenital heart disease (CHD) is the most common birth defect with various incidence of 4‐50 per 1000 live births, worldwide,^1^ consisting different types of cardiac malformations from severe forms such as tetralogy of Fallot (TOF) and transposition of the great arteries (TGA) to mild forms such as bicuspid aortic valve (BAV).^2^ Although 8% of CHD cases are estimated to result from chromosomal abnormalities and single gene disorders^3^, the exact etiology of CHD is mostly unknown (about 75% of cases).^4^ Previous studies indicated some transcription factors which play significant role in heart development including GATA binding protein 4 (*GATA4*), *NOTCH*, myosin heavy chain 6 (*MYH6*), NK2 homeobox 5 (*NKX2‐5*), Zic family member 3 (*ZIC3*), T‐box 5 (*TBX5*), and T‐box 20 (*TBX20*).^5^, ^6^, ^7^

Among these transcription factors, *GATA4*, located on 8p23.1, is expressed in endocardial, myocardial, and mesenchymal cells for suitable cardiac septation.^8^ As a critical zinc finger transcription factor, it is important in different types of CHD causality, that is, TOF, ventricular septal defect (VSD), atrioventricular septal defect (AVSD), atrial septal defect (ASD), patent ductus arteriosus (PDA), and pulmonary valve stenosis (PS).^5^, ^6^, ^9^, ^10^ Functional experiments in different animal models such as mice, fly, and fish demonstrated that any change in *GATA4* sequence can affect cardiac development.^11^, ^12^ Full length of *GATA4* cDNA is 3371 bp, and it contains 6 exons. The GATA4 protein has 442 amino acids and binds to the GATA motif of target genes involved in cardiogenesis.^13^ To date, based on Human Gene Mutation Database (HGMD) (www.hgmd.cf.ac.uk), 114 mutations in *GATA4* gene have been reported in the CHD cases.

Given there are few studies^14^, ^15^, ^16^ which surveyed CHD etiology in Iranian population, we evaluated the entire coding sequence of *GATA4* gene in sixty‐six familial CHD patients. This study is the first report of a clinical significance of a pathogenic *GATA4* mutation in an Iranian patient with BAV. Also, this study is the first report of a novel balanced translocation^5^, ^7^ that may or may not related with heart defect phenotype in the same patient.

## 2 MATERIALS AND METHODS

### 2.1 Study subjects and samples

Sixty‐six patients affected from any types of CHD, as confirmed by echocardiography, who had been referred to the Rajaei Heart Center (Tehran, Iran) between November of 2015 and the end of 2017 as well as some of their family members (from fifty‐five pedigrees) were enrolled in this study. A comprehensive genetic counseling was performed for all families. We previously investigated the genetic cause of CHD in these patients using sequence analysis of the *NKX2‐5* 17, *ZIC3*, *NODAL*, *FOXH1*, *GJA1* genes, and MLPA (multiplex ligation‐dependent probe amplification) for common reported deletions and array CGH (array comparative genomic hybridization) for genomic imbalances; no change was detected. In this study, karyotype analysis, whole‐exome sequencing (WES) in the case (CHD‐7) that we found structural changes in his chromosomes, and *GATA4* variant were checked; briefly, peripheral blood from all the patients was collected in heparin and EDTA tubes, both. Heparinized blood was used for karyotyping, based on the standard protocols as described previously^18^, and EDTA‐treated samples for DNA extraction. The study is performed in accordance with the Helsinki Declaration and has been approved by the Rajaei Cardiovascular, Medical, and Research Center (RHC.AC.IRREC.1395.46; December 24, 2016) and Zanjan University of Medical Sciences (ZUMS.REC.1396.145; June 21, 2017) Ethics Committees.

### 2.2 Primers, PCR, and direct Sanger sequencing

Five millilitre peripheral blood from each subject was collected in EDTA‐containing tubes, and genomic DNA was extracted according to our in‐house method based on the standard salting‐out technique. To amplify the entire *GATA4* coding sequence as well as exon‐intron boundaries, six primer pairs were designed (Table S1). Polymerase chain reaction (PCR) was then performed on a SimpliAmp™ Thermal Cycler (Thermo Fisher Scientific) with 100 ng genomic DNA (gDNA), 10 pmol/L primers, 200 mmol/L dNTP, 1.5 mmol/L MgCl_2_, and 1 U of Taq DNA polymerase (Amplicon, UK). After incubation of the entire mix at 95°C for 5 minutes, 35 amplification cycles (40 seconds at 95°C, 30 seconds at 60°C, and 30 seconds at 72°C) were performed. PCR products were then subjected to Sanger sequencing‐based analysis on an ABI Sequencer 3500XL PE (Applied Biosystems) using the same primer sets. The sequences were subsequently analyzed using FinchTV 1.4.0 (www.geospiza.com/finchTV) and visually evaluated for polymorphisms. Variants were named based on the Human Genome Variation Society (HGVS) nomenclature.^19^

### 2.3 Whole‐exome sequencing

DNA sample of candidate case (CHD‐7) was subjected to WES at Macrogen (Seoul, South Korea). 10 ng of DNA was applied for exome enrichment by SureSelect XT Library Prep Kit. WES was performed on an Illumina HiSeq 4000 according to the manufacturer's protocol (Illumina) and generating paired‐end reads, that is, read quality of >20 and depth of >5 were applied for further analyses.

It should be noted that the overall coverage of the whole exome (read depth 1X) was 99.8% but this was only 68.4% coverage with a read depth of 50X, more than 90% reads have average Phred scores above 20, the mean of per sequence quality scores was more than 36, per sequence GC content was ~59%, and the sequence length distribution was about 150 bp.

The generated sequences were aligned with the human reference genome (NCBI build37/hg 19 version) using Burrows‐Wheeler Aligner (BWA) (http://bio-bwa.sourceforge.net/).^20^ The variants were called by Genome Analysis Toolkit (GATK) (https://www.broadinstitute.org/gatk/)^21^ and annotated with ANNOVAR (http://annovar.openbioinformatics.org/).^22^ The variant frequency filtering was performed using 1000 Genomes Project (http://www.1000genomes.org/), Exome Aggregation Consortium (ExAC) (http://exac.broadinstitute.org/), and dbSNP (https://www.ncbi.nlm.nih.gov/projects/SNP/). Minor allele frequency (MAF) of 0.0005 for dominant and 0.005 for recessive variants were considered; in addition, synonymous changes and variants which were out of exonic sequences were excluded. ACMG Standards were used for the interpretation of sequence variants.^23^

### 2.4 Bioinformatics

Candidate variants were evaluated for their potential effect(s) on protein function and structure of GATA4 using bioinformatic prediction tools such as Mutation Taster (http://www.mutationtaster.org/),^24^ sorting intolerant from tolerant (SIFT) (http://sift.bii.a-star.edu.sg/),^25^ combined annotation–dependent depletion (CADD) (http://cadd.gs.washington.edu/home),^26^ and polymorphism phenotyping v2 (PolyPhen‐2) (http://genetics.bwh.harvard.edu/pph2/).^27^ We also used CLUSTALW (https://www.genome.jp/tools-bin/clustalw)^28^ for multiple alignment of GATA4 protein sequence in human as compared with other organisms. The potentially pathogenic variants were then traced inside the pedigree using PCR‐based Sanger sequencing.

## 3 RESULT

### 3.1 Cytogenetic analysis

Cytogenetic analysis of the patients for genome and chromosomal mutations appeared normal in all, but one, the CHD‐7, for whom G‐banding revealed a novel balanced translocation between chromosomes 5 and 7 (46,XY,t(5;7)(qter13;qter11)) in 20 metaphase spreads that were studied. Segregation analysis showed the absence of that translocation in both parents. To investigate the presence of any other pathogenic genomic mutation linked to CHD, WES was performed on the CHD‐7 patient. Subsequently, a heterozygous c.1309G>A (p.Gly437Arg) variation in GATA4 coding sequence was identified. This case is further described below.

### 3.2 De novo balanced 46,XY,t(5;7)(qter13;qter11) translocation does not seem to be causative for CHD

**Figure 1:**
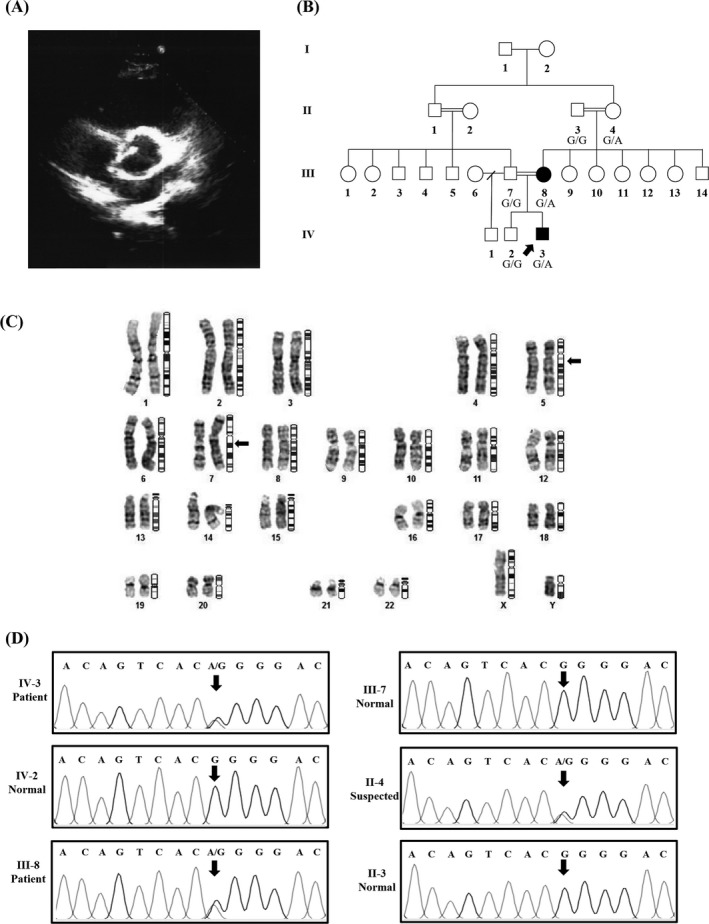
Clinical and *GATA4* genetic analysis of the CHD‐7 patient. A, Echocardiogram of the patient having bicuspid aortic valve (BAV). B, The CHD‐7 patients in which the proband (IV‐2) and his affected mother (III‐8) are indicated with filled symbols. The proband's grandmother (II‐4) was suspected for CHD but did not agree with further examination. C, The G‐banded karyotype of the CHD‐7 patient. Arrow is pointing to the position of the identified t(5;7)(qter13;qter11) balanced translocation. D, Partial chromatograms show the results of *GATA4* exon 7 sequencing in CHD‐7 patient (IV‐2), proband's mother (III‐8), father (III‐7), brother (IV‐3), grandmother (II‐4), and grandfather (II‐3) among whom IV‐2, III‐8, and II‐4 individuals are heterozygous carriers of the c.1309G>A variant in *GATA4*

A 14‐year‐old boy to whom we are referring as CHD‐7 throughout the study was admitted to our hospital suffering from shortness of breath. Clinical examination and echocardiography unraveled the presence of BAV, as shown in Figure 1A. Through genetic counseling, the history of CHD was confirmed in the pedigree. Proband's mother (III‐8 in Figure 1B), a 40‐year‐old lady, had been diagnosed with ASD at the age of 28. Her 62‐year‐old mother (II‐4 in Figure 1B) was also suffering from the shortness of breath, started when she was 50 years old. However, she did not agree to participate in this study.

As stated before, 46,XY,t(5;7)(qter13;qter11) balanced translocation identified in the pedigree's proband (CHD‐7 patient) was neither found in his affected mother nor in his healthy father (Figure 1A). However, the heterozygous c.1309G>A (p.Gly437Arg) variation (rs748737164) identified in the proband (CHD‐7 patient) by WES was detected in his affected mother (III‐8 in Figure 1B), as expected. None of the other family members who were studied including II‐3, III‐7, and IV‐3 individuals (Figure 1B) carry that variation. No information on the frequency of this variation could be found in Iranome, ExAC (The Exome Aggregation Consortium), 1000 Genome, and TOPMED (The Trans‐Omics for Precision Medicine) (Table 1). The novel c.1309G>A variant was detected in one patient through WES. The scores of 32 in CADD, 0.00 in SIFT, and 0.99 in Polyphen‐2 as well as in silico analysis by Mutation Taster tool potentially propose this variant as missense mutation.

**Table 1:** The *GATA4* variants in this study

Gene	dbSNP	Variant	Gene region	Variant type	Amino acid change	Hetero‐/homozygote	Variant frequencies (%)				
							Current study	Iranome	ExAC	1000 Genome	TOPMED
*GATA4*	rs3779664	c.783+454G>A	Intron 3	Polymorphism	−	0/17	26.1	−	−	0.1837	0.1333
	rs550991623	c.909+20A>G	Intron 4	Polymorphism	−	5/0	7.7	−	0.0000	0.0002	0.0000
	rs804280	c.997+56C>A	Intron 5	Polymorphism	−	37/15	80.0	0.6256	−	0.2656	0.3778
	rs11987175	c.997+287A>C	Intron 5	Polymorphism	−	12/6	27.7	−	−	0.3624	0.3034
	rs3729851	c.997+200G>A	Intron 5	Polymorphism	−	12/2	21.5	−	−	0.0575	0.0906
	rs3729856	c.1129A>G	Exon 6	Polymorphism	p.Ser377Gly	23/7	46.1	0.1256	0.0962	0.0429	0.0819
	rs114868912	c.1138G>A	Exon 6	Polymorphism	p.Val380Met	6/0	9.2	0.0012	0.0063	0.0156	0.0116
	rs867858	c.*354A>C	3'UTR	Polymorphism	−	26/13	60.0	−	−	0.3614	0.3043
	rs1062219	c.*426C>T	3'UTR	Polymorphism	−	32/17	75.4	−	−	0.2272	0.3215
	rs115099192	c.1220C>A	Exon 7	Mutation	p.Pro407Gln	2/0	3.0	0.0006	0.0006	0.0012	0.0002
	−	c.*100G>A^a^	3'UTR	Polymorphism	−	1/0	1.5	−	−	−	−
	rs748737164	c.1309G>A^b^	Exon 7	Mutation	p.Gly437Arg	1/0	1.5	−	−	−	−

Note

This table provides all information about identified variants in this study including gene name, dbSNP rs (The Single Nucleotide Polymorphism Database reference SNP), DNA sequence change, position of the variant, variant type (mutation or polymorphism), protein sequence change, genotype, frequency of variants in our study, Iranome, ExAC (The Exome Aggregation Consortium) databases, 1000 Genome, and TOPMED (The Trans−Omics for Precision Medicine) projects.

a Novel variant.

b First clinical report of this variant. Minus (−) means there is no information.

### 3.3 Distribution of *GATA4* variations among Iranian CHD patients

66 CHD patients, numbered as CHD‐1 to CHD‐66 (our information about individuals condition was based on genetic counseling and family report), were recruited in this study in which no previously known CHD‐related pathogenic variant was found in the *NKX2‐5*, *ZIC3*, *NODAL*, *FOXH1,* and *GJA1* genes by PCR‐direct sequencing. Out of 66, VSD was the most frequent CHD (40.9%) followed by TOF in 25.8% and PDA in 16.7%. The other CHD frequency was ASD (15.1%), pulmonary stenosis (PS) (7.6%), TGA (7.6%), double outlet right ventricle (DORV) (4.5%), AVSD (3%), coarctation of the aorta (COA) (1.5%), hypoplastic left heart syndrome (HLHS) (1.5%), and BAV (1.5%). The outcome of *GATA4* variant screening is summarized in Table 1, and CHD types in our studied population are indicated in Table S2.

The c.783+454G>A transition was detected as homozygous within the intron 3 of the gene in 17 patients. In the fourth intron, a c.909+20A>G transition was identified in 5 heterozygotes. The c.997+56C>A, c.997+287A>C, and c.997+200G>A variants of the fifth intron were found in various numbers of patients as heterozygotes and homozygotes, both (Table 1). Comparing frequency of all those intronic variants in several databases including 1000 Genome, TOPMED, and ExAC confirmed them as genetic polymorphisms with the c.997+56C>A as the most frequent benign variation identified in the studied population.

Some other benign variations were also found within the 3'UTR of *GATA4* gene including the heterozygote c.*354A>C variant in 39 patients, the homozygote c.*426C>T variants in 49 patients, and the heterozygote c.*100G>A observed in one patient. The novel c.*100G>A variant was found in a 12‐year‐old boy with tetralogy of Fallot (TOF). The presence of that variant in the patient's healthy mother upon segregation analysis confirmed that it is benign.

The homozygotes c.1129A>G in 30 patients and c.1138G>A in 6 patients, both located within the exon 6, were also identified. The c.1129A>G changes serine to glycine, and the c.1138G>A changes valine to methionine. These polymorphisms are documented as high frequent variants with no pathogenic effects according to the current databases and previous reports.^29^ However, two identified missense mutations, the c.1309G>A and the c.1220C>A within the exon 7, seem to be related to the observed phenotype.

**Figure 2:**
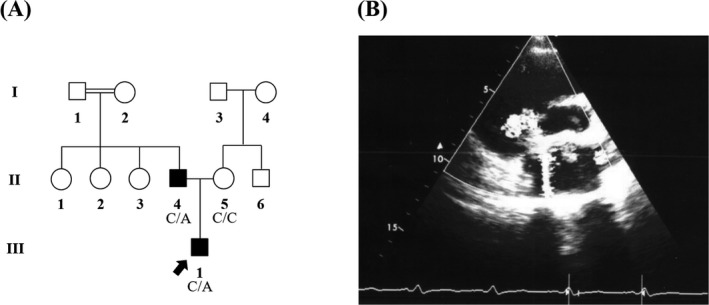
*GATA4* genetic and clinical analysis of the CHD‐33 pedigree. A, The family pedigree in which proband at the age of 6 months old (III‐1) displayed ventricular septal defect (VSD); also, his father (II‐5) was affected with VSD. B, Presents echocardiogram of the patient (III‐1) with VSD. III‐1 and II‐5 patients indicated c.1220C>A mutation as heterozygous form

The heterozygote c.1220C>A was identified in two affected individuals from a single pedigree. The C>A transversion substitutes proline with glutamine at codon 407 (p.Pro407Gln) of GATA4. This previously reported missense mutation was observed in a 6‐month‐old boy and his 25‐year‐old father both suffering from ventricular septal defect (VSD) (Figure 2B). Both patients also harbored the c.997+56C>A and c.1138G>A variants which have been reported as genetic polymorphisms.

## 4 DISCUSSION

Sixty six CHD patients of various ethnicities within the Iranian population were screened in this study. Since our center is one the major country's referral hospitals for cardiogenetic diseases, the studied patients were of diverse ethnicities including Fars, Azeri, Kurd, Lur, Gilaki, Mazandarani, Arab, Turkmen, and Baloch. The presence of the disease in the patient was confirmed by expert cardiologists.

G‐banding screening of all the patients resulted in identification of a de novo balanced chromosomal translocation, t(5;7)(qter13;qter11), in a 14‐year‐old boy with BAV. None of his parents including the mother who is suffering from ASD carry the translocation. Using WES data for CHD‐7, we tried to identify chromosomal breakpoint locations but we failed.^30^ In addition, no CHD‐related CNV was identified throughout the chromosomes 5 and 7, as far as our WES data could have provide. Through WES of the mentioned patient (CHD‐7), a heterozygous c.1309G>A (p.Gly437Arg) variation in *GATA4* (one of the important CHD‐associated genes) was identified to which no defined phenotype has been correlated. The p.Gly437Arg mutation in our patient is predicted likely pathogenic based on most amino acid change predictors such as CADD (score: 32). It should be noted that another mutation at the same base, c.1309G>T (p.Gly437Trp), is described in ExAc with predicted pathogenicity. The presence of the variant was confirmed in the patient's mother and grandmother who seem to be affected from a different types of CHD. Such a phenotypic difference could raise the hypothesis that the patient's phenotype may have been modified by the presence of the chromosomal translocation. Though we stopped further investigation due to the unwillingness of the family to further participate in the study, this chromosomal abnormality should be noted in genetic counseling as individuals with balanced chromosomal translocations might have fertility difficulties such as infertility or giving birth to babies with congenital anomalies, despite normal phenotypes.^31^

To the best of our knowledge,^16^, ^32^, ^33^ the *GATA4* gene can be considered as one of common genes involved in CHD etiology. We screened the entire coding sequence regions of the *GATA4* gene among sixty‐six CHD patients. Except for one, other pathogenic variant, the c.1220C>A observed in one family (2 cases), the rest were benign genetic polymorphisms that have been reported in some other populations, too.

**Figure 3:**
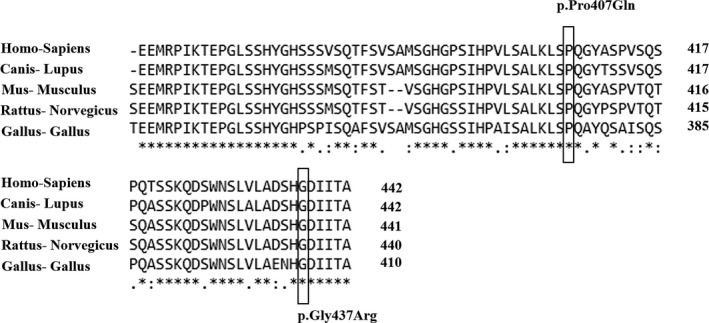
Multiple sequence alignment of the GATA4 protein sequences in Homo sapiens (human, NP_002043), Canis lupus (dog, NP_001041577), Mus musculus (mouse, NP_032118), Rattus norvegiocus (rat, NP_653331), and Gallus gallus (chicken, XP_420041) was performed using the CLUSTALW version 2.1. The missense mutation positions are boxed. Missense mutations are highly conserved positions

GATA4 as a conserved transcription factor regulates over 30 genes that are signaling pathway players of the heart development.^34^ GATA4 has two transcriptional activation domains (TAD), N‐terminal zinc finger (NZF) and C‐terminal zinc finger (CZF).^35^ Locations of the two detected missense mutations, the p.Gly437Arg and p.Pro407Gln, in the protein structure are illustrated in Figure 3. The c.1309G>A mutation changes glycine to arginine residue at 437 position. In silico analyses confirmed that the altered amino acid in protein sequence was highly conserved among different species (Figure 3), and this mutation was predicted to be damaging.

To our knowledge, this study is the first report on the c.1309G>A clinical manifestation. In addition, here the c.1220C>A mutation results in p.Pro407Gln, has been linked to VSD. Similar to our observation, Peng et al^36^ have reported c.1220C>A causing VSD in CHD patients. The c.1309G>A and c.1220C>A are both occur in the C‐terminal region of GATA4. The C‐terminal domain interacts with the *HAND2*, *NF‐AT3*, *MEF2C,* and *NKX2.5* transcription factors which play important roles in the heart development.^37^ Although two other variants, c.1129A>G and c.1138G>A, were also detected in the conserved regions, in silico and segregation analyses, that is, the present variants in the healthy members of the families, indicated these changes have no effect in CHD manifestation. Schluterman et al^29^ and Hamanoue et al^38^ detected c.1138G>A in their studied patient/control groups that has no effect in the CHD pathology. c.1129A>G was also reported previously in normal controls as well as CHD patients in some documents.^29^, ^39^, ^40^

Variable expressivity has been reported in GATA4‐causing CHDs.^41^ In a study by Tomita‐Mitchell et al,^9^ carriers of c.278G>C, c.946C>G, c.1232C>T, and c.1273G>A mutations presented ASD, VSD, and TOF. Similarly, we observed the presence of BAV in a carrier of the heterozygous c.1309G>A mutation (the CHD‐7 family) while his mother with the same mutation only had ASD. Although in this case, the potential effect of the balanced 46,XY,t(5;7)(qter13;qter11) translocation cannot be fully rejected.

In conclusion, here we report for the first time a de novo balanced translocation may or may not related to the CHD phenotype. We also present c.1309G>A as the BAV causing likely pathogenic mutation in *GATA4* gene. As mentioned before, no clinical evidence for that mutation has been presented so far. Correlating *GATA4* mutations to phenotypes in congenital BAV, ASD, and VSD provides a ground for early diagnosis of these defects in families who have affected individuals.

## AUTHORS' CONTRIBUTIONS

SK, TSH, and NM wrote the article. SK carried out the experiments. NM, AB, MM, and HA contributed to patient's diagnosis. HRZ and MCA performed computational analysis of the data. SK, TSH, and NM contributed to project management, genetic analyses, interpretation of data, revision of the initial manuscript, and final approval.

## ETHICAL APPROVAL

Informed consent has been obtained by the authors.
