# Supplementary material for: GATA4 screening in Iranian patients of various ethnicities affected with congenital heart disease: Co‐occurrence of a novel de novo translocation (5;7) and a likely pathogenic heterozygous GATA4 mutation in a family with autosomal dominant congenital heart disease
Source: J Clin Lab Anal. 2019 May 22;33(7):e22923. doi: 10.1002/jcla.22923 (PMC6757118; doi:10.1002/jcla.22923)
Supplement: Supplementary file 1 [file JCLA-33-e22923-s001.docx]

**Supplementary Table 1.** Primers designed to amplify the entire *GATA4* coding sequence.

| **Primers** | **Sequence 5’-3’** | **Nucleotide position** | | **Product size(bp)** |
| --- | --- | --- | --- | --- |
| **Exon 2** | F:TGAAAGCTCTGGGATGAACC  R:AACAAGAGGCCCTCGACAG | c.-120(Intron1) to c.-101(Intron1)  c.616+47(Intron2) to c.616+67(Intron2) | | 1202 |
| **Exon 3** | F:CATTGTTTCTGTGCGCTCTAG  R:GTGGCTCCAGCTAACTCTAAAG | c.783-268(Intron2) to c.783+248(Intron2)  c.783+143(Intron3) to c.783+164(Intron3) | 433 | |
| **Exon 4** | F:AGAGATCTCATGCAGGGTCG  R: GAGGACTGAGAGATGGGCAT | c.909-261(Intron3) to c.909-242(Intron3)  c.909+41(Intron4) to c.909+60(Intron4) | | 321 |
|  |  |  |  |  |
|  |  |  |  |  |
| **Exon 5** | F:GCAGCAGGTGTGTGTCTTTC  R:CCAAAGATGAAAGGACCGAG | c.997-168(Intron4) to c.997-149(Intron4)  c.997+300(Intron5) to c.997+319(Intron5) | | 487 |
| **Exon 6** | F:GGCTGTTCGTTTGTCCCTG  R:TCAATGGCTGGGTCTTCCTA | c.1146-205(Intron5) to c.1146-187(Intron5)  c.1146+132(Intron6) to c.1146+151(Intron6) | | 356 |
| **Exon 7** | F:AAGTGCTCCTTGGTCCCTT  R:CTATGTGTGACACGGTGAACG | c.1329-320(Intron6) to 1329-302(Intron6)  c.1329+807(Intron7) to c.1329+827(Intron7) | | 1147 |

Primers were designed against *GATA4* reference sequence with the Accession No. NG_008177.2

**Supplementary Table 2.** CHD types in the study population.

| **Family No.** | **Patient No.** | **CHD** | **Family No.** | **Patient No.** | **CHD** |
| --- | --- | --- | --- | --- | --- |
| 1 | 1 | ASD | 29 | 34 | AVSD/PDA |
| 2 | 2 | VSD |  | 35 | VSD |
| 3 | 3 | DORV | 30 | 36 | ASD |
| 4 | 4 | TOF | 31 | 37 | TOF |
| 5 | 5 | ASD/VSD |  | 38 | TOF |
| 6 | 6 | ASD | 32 | 39 | HLHS |
|  | 7 | BAV | 33 | 40 | VSD |
| 7 | 8 | TGA/PDA | 34 | 41 | VSD |
| 8 | 9 | TOF |  | 42 | VSD |
| 9 | 10 | ASD/PDA | 35 | 43 | PDA |
| 10 | 11 | VSD/PA | 36 | 44 | TGA |
| 11 | 12 | ASD/PS | 37 | 45 | PS |
| 12 | 13 | VSD/PS/DORV |  | 46 | ASD |
|  | 14 | VSD | 8 | 47 | PDA |
| 13 | 15 | TOF | 39 | 48 | VSD |
| 14 | 16 | PDA | 40 | 49 | ASD |
| 15 | 17 | TGA | 41 | 50 | TOF |
| 16 | 18 | TOF |  | 51 | VSD/PS |
| 17 | 19 | VSD/PDA/COA | 42 | 52 | TOF |
|  | 20 | VSD | 43 | 53 | TOF |
| 18 | 21 | TOF | 44 | 54 | VSD |
| 19 | 22 | TOF |  | 55 | VSD |
| 20 | 23 | VSD/PDA | 45 | 56 | TOF |
| 21 | 24 | VSD | 46 | 57 | VSD/PS |
|  | 25 | VSD | 47 | 58 | AVSD/PDA |
| 22 | 26 | TGA | 48 | 59 | ASD |
| 23 | 27 | PDA | 49 | 60 | TOF |
| 24 | 28 | TOF | 50 | 61 | TGA |
| 25 | 29 | VSD | 51 | 62 | TOF |
| 26 | 30 | VSD | 52 | 63 | VSD/DORV |
| 27 | 31 | TOF | 53 | 64 | ASD/PDA |
| 28 | 32 | VSD | 54 | 65 | TOF |
|  | 33 | VSD | 55 | 66 | VSD |

TGA, [Transposition of the Great Arteries](http://www.stanfordchildrens.org/en/topic/default?id=transposition-of-the-great-arteries-tga-90-P01823); DORV, [Double Outlet Right Ventricle](https://www.rch.org.au/cardiology/heart_defects/Double_Outlet_Right_Ventricle/); TOF, [Tetralogy of Fallot](https://www.cincinnatichildrens.org/health/t/tof); [Total Anomalous Pulmonary Venous Return](https://www.cincinnatichildrens.org/health/t/tapvr); ASD, [Atrial Septal Defect](http://www.heart.org/HEARTORG/Conditions/CongenitalHeartDefects/AboutCongenitalHeartDefects/Atrial-Septal-Defect-ASD_UCM_307021_Article.jsp); AVSD, [Atrioventricular septal defect](https://en.wikipedia.org/wiki/Atrioventricular_septal_defect); VSD, [Ventricular Septal Defect](http://www.heart.org/HEARTORG/Conditions/CongenitalHeartDefects/AboutCongenitalHeartDefects/Ventricular-Septal-Defect-VSD_UCM_307041_Article.jsp); HLHS, [Hypoplastic Left Heart Syndrom; PS, [Pulmonary Stenosis](https://www.rch.org.au/cardiology/parent_info/Pulmonary_Stenosis_PS/); PA, Pulmonary artery stenosis; AS, Aortic stenosis; COA, Coarctation of the aorta; PDA, [Patent Ductus Arteriosus](http://www.heart.org/HEARTORG/Conditions/CongenitalHeartDefects/AboutCongenitalHeartDefects/Patent-Ductus-Arteriosus-PDA_UCM_307032_Article.jsp); BAV, [Bicuspid aortic valve](https://en.wikipedia.org/wiki/Bicuspid_aortic_valve)](https://www.rch.org.au/cardiology/parent_info/Hypoplastic_Left_Heart_Syndrome/)
